# Formulation of Polymeric Microparticles Using Eco-Friendly Extracted Crude Fucoidans from Edible Brown Seaweed *Undaria pinnatifida*

**DOI:** 10.3390/foods12091859

**Published:** 2023-04-29

**Authors:** Tania Ferreira-Anta, Maria Dolores Torres, Herminia Dominguez, Noelia Flórez-Fernández

**Affiliations:** CINBIO, Department of Chemical Engineering, Campus Ourense, Edificio Politécnico, Universidad de Vigo, As Lagoas, 32004 Ourense, Spain; tania.ferreira@uvigo.es (T.F.-A.); matorres@uvigo.es (M.D.T.); noelia.florez@uvigo.es (N.F.-F.)

**Keywords:** antioxidant activity, alginate, biopolymer, encapsulation, rheological properties, pressurized hot water extraction

## Abstract

Several bioactive compounds that hold a potential interest in the food industry as phenolic compounds, polysaccharides, proteins and vitamins, among others, are present in seaweeds. Green extraction technologies are the preferred way to obtain these compounds. Pressurized hot water extraction, from 160 to 220 °C, was tested to achieve high yields of these components from the edible brown seaweed, *Undaria pinnatifida*. The maximum fucoidan content was recovered at 160 °C, while the phloroglucinol content and antioxidant activity were maximum at 220 °C. The possibility of encapsulating these bioactive fractions using mannitol was assessed. The highest production yield of the polymeric particles was found using the 220 °C fraction (close to 75%). In order to formulate microparticles with bioactive potential, several ratios of liquid phases were assessed, 3:1, 1:1 and 1:3 (*w*:*w*), using the liquid fractions obtained at 160 °C and 220 °C. The yield production was always above 67%, being in the 1:3 ratio (160 °C:220 °C) and close to 75%. The rheological results indicated that the presence of microparticles enhanced the apparent viscosity of the aqueous dispersions with non-Newtonian profiles, achieving the highest viscosity for those formulated with microparticles from 160 °C:200 °C (3:1).

## 1. Introduction

Different marine seaweeds have different and exclusive bioactive compounds with potential applications in several sectors, such as food, nutraceuticals and cosmetics [1,2]. Depending on their color, seaweeds are classified as green, red or brown based on their main pigments, chlorophyll, phycoerythrin and fucoxanthin, respectively. Moreover, their major polysaccharides (ulvan, carrageenan and fucoidan, respectively) have very different properties and characteristics, generating attractive potential uses and commercial products [3,4,5].

In the last years, seaweeds have gained attention for their multiple nutraceutical properties due to the composition of the algae having a high nutritional value and bioactivity. Proteins, carbohydrates, phenolic compounds and others are essential in the human diet [6]. Furthermore, the biological properties, such as antioxidant [7], antitumoral, anti-inflammatory [8], or antimicrobial properties, have boosted studies using these raw materials, which are sometimes under-used. The main characteristic of brown seaweeds is fucoidan, a polysaccharide comprising fucose, galactose, mannose, xylose, arabinose, glucose uronic acids and sulphate groups. Focusing on the raw material of the work, *Undaria pinnatifida* is a brown seaweed that is widely distributed due to its invasive character [9]. It is an edible seaweed with a high presence in the sea and oceans, with an interesting composition applicable to the food field [10].

The aforementioned compounds (fucoidan, phenolics, proteins, etc.) are in the cell wall of the seaweed, and in order to achieve their release, an adequate extraction methodology strategy is required [10,11,12,13]. Conventional extraction technologies have some advantages, such as simplicity, but their disadvantages, such as low yield of some compounds or larger times of extraction, have led to exploring other possibilities. Eco-friendly alternatives, such as ultrasound-assisted extraction, microwave-assisted extraction or subcritical water extraction, are more attractive technologies for achieving higher yields of extraction [5,14]. These treatments allow the use of water as an extracting agent with a short operation time.

The formulation of microparticles for applications in the food industry, biomedicine and cosmetics has been explored [15,16,17]. In this context, the encapsulation of bioactive compounds promotes several benefits; one example is the protection of sensitive elements or substances from oxidation [15]. This special feature is very attractive in terms of application, being able to extend the useful life of a product. Among the numerous materials suitable as carriers, mannitol is one of the most used in the pharmaceutical field due to its properties [18].

The main aim of this work was to produce polymeric particulate systems from extracts obtained by pressurized hot water extraction from *U. pinnatifida*. As secondary goals, we studied the characterization of the liquid extracts, alginates and the behavior of microparticles, focusing on the suitable characteristics to apply in the food industry.

## 2. Materials and Methods

### 2.1. Analysis of Raw Material

*Undaria pinnatifida* brown seaweed was purchased from Algamar (Pontevedra, Spain). The proximal composition regarding the moisture, ash, protein, sulphate, acid-insoluble residue, carbohydrate content and minerals was published in previous work (ash: 38%, carbohydrates: 17%, sulphate: 3%, protein: 15%, acid-insoluble residue: 24% and minerals with sodium and potassium as the main compounds), where the solid residue fraction was also evaluated [19].

### 2.2. Extraction Process

#### Pressurized Hot Water Extraction

The dried and milled brown seaweed was mixed with distilled water at a solid–liquid ratio of 1:30 (*w*/*w*) in a stainless steel pressurized and stirred reactor (Parr Instruments, series 4848, Moline, IL, USA). Following previous works performed by the group [20], the selected extraction temperatures were 160, 180, 200 and 220 °C. The pressure of the treatment was approximately 110 psi (around 7.5 atm). After extraction, the fractions obtained (liquid and solid) were separated by vacuum filtration (GM-0.50, Comecta, Spain) and characterized. The severity of the extraction process, calculated following the equation previously proposed [21], ranged between 1.50–4.00. 

### 2.3. Solid Residues Fractions Characterization

The fractions obtained after the corresponding experiment at different extraction temperatures were separated by vacuum filtration, dried at room temperature and characterized. The moisture content of the samples was analyzed following a gravimetric method (Association of Official Analytical Chemists, 2000). After 48 h in an air convective oven at 105 ± 2 °C (JP Selecta, Barcelona, Spain), the content of water in the samples was analyzed. The ash content was also determined gravimetrically in a muffle furnace (ELF, Carbolite, UK) at 575 °C for 6 h. The Kjeldahl method was used to determine the protein content by applying the conversion factor (5.38) according to previous work [22]. 

#### Minerals

The Na, K, Ca, Mg, Fe, I, P, Zn, Cd, Cu, C and H elements were determined by several methods previously described [19,20]. In addition, C and H were also analyzed in order to determine the high heating value (HHV) of the solid residue after extraction [23]. These values were estimated for the residual solid obtained at all the temperatures following Equation (1):

HHV (kJ/kg) = 3.55·C^2^ − 232·C − 2230·H + 51.2·C∙H + 131·N + 20600 (1)

### 2.4. Liquid Fractions Characterization

The soluble extract was analyzed to determine the dry matter content, and the sample (1 mL) was dried at 105 °C for 48–72 h in an air convective oven (P Theroven, JP Selecta, Barcelona, Spain). The pH value was measured using a pH meter (Crison GLP 21, Crison, Barcelona, Spain), and standard liquids from Crison were used to calibrate the equipment.

#### 2.4.1. Protein Content 

The Bradford method was used to evaluate the content of soluble protein [24]. Bovine serum albumin, or BSA (Sigma, St. louis, MO, USA), was used as a standard. The protocol indicated by the supplier of the Bradford reactive (Sigma, St. louis, MO, USA) was performed, and the absorbance was measured at 595 nm.

#### 2.4.2. Antioxidant Features 

The phloroglucinol content was measured following the protocol described by Koivikko et al. (2005) [25]. Briefly, the liquid sample (the soluble extract from the pressurized hot water extraction) or a blank (1 mL) was placed in a test tube, and the Folin Ciocalteu reagent (1 N) and Na_2_CO_3_ (20%) were then added, 1 and 2 mL respectively. The test tubes were stirred (vortex) and incubated in darkness (45 min at room temperature). Phloroglucinol (Sigma-Aldrich, Madrid, Spain) was used to prepare the standard curve. The absorbance was read at 730 nm (Evolution 201 UV–vis, Thermo Scientific, Waltham, MA, USA).

The trolox equivalent antioxidant capacity (TEAC) assay was performed to evaluate the antioxidant capacity [26]. The liquid samples obtained from the extraction and PBS as blank (20 μL), were placed in a test tubes, and 2 mL of ABTS solution (adjusted to absorbance 0.7 ± 0.1 at 734 nm), was added. The test tubes were incubated in a water bath (6 min, 30 °C), and the absorbance was measured at 734 nm in triplicate. Trolox (ACROS organics, Slovakia) was the standard used to perform the standard curve. Data were expressed as TEAC values. 

DPPH (α,α-diphenyl-β-picrylhydrazyl) or radical scavenging activity was evaluated for the liquid samples or extracts obtained after eco-friendly extraction [27]. The extracts were placed in test tubes (50 μL) and 2 mL of the DPPH solution (absorbance = 0.6 ± 0.1, at 515 nm) and were mixed. The IC_50_ was expressed as g/L.

#### 2.4.3. Oligosaccharide Determination

The samples were dialyzed using a tubing membrane (Spectra/Por Float-A-Lyzer G2 Dialysis Membrane Tubing, MWCO 0.5 kDa, Spectrum Labs) to remove the monosaccharide fraction. After that, the liquid samples were mixed with sulfuric acid at 4% (*w*/*w*) in suitable flasks and introduced in an autoclave (121 °C, 20 min, 2 atm) in order to hydrolysate the oligo and polymeric compounds to the constituent monomers [28]. Next, the samples were filtered by 0.45 µm prior to being placed in a vial for the HPLC measurements. High-performance liquid chromatography (HPLC, 1260 Infinity, Agilent Technologies, Santa Clara, CA, USA) was used to determine the oligosaccharide fraction using the following standards: glucose, galactose, mannose, fucose, acetic acid and glucuronic. The columns used for this determination were described elsewhere [29], and the quantification was expressed as oligosaccharides.

#### 2.4.4. Sulfate Content

The determination of the sulfate content was performed using the gelatine-barium chloride method [30]. The gelatin-BaCl_2_ reagent was previously prepared: 0.5 g of gelatine powder (Scharlau, Barcelona, Spain) was placed in a beaker, and hot water (100 mL) was added. Next, the solution was kept at 4 °C for at least 6 h, following 0.5 g of BaCl_2_ (Scharlau, Barcelona, Spain) being added, obtaining a cloudy solution; 2–3 h later, the solution was ready to use. A volume of 0.1 mL of the samples was placed in a test tube, and 1.9 mL of trichloroacetic acid at 4% (*w*/*v*, Scharlau, Barcelona, Spain) was added. Additionally, 1 mL of gelatine-BaCl_2_ reagent was added, mixed and incubated at room temperature for 15 min. The absorbance was measured at 500 nm.

All the above analyses were performed at least in triplicate.

### 2.5. Structural Profiles

#### 2.5.1. High-Performance Size Exclusion Chromatography

The distribution of the molar mass profiles of the extracts from *U. pinnatifida* oligosaccharides found in the soluble fractions was assessed by high-performance size-exclusion chromatography. The aforementioned HPLC was used with a TSKGel SuperMultipore PW-H (6 × 150 mm) column with a TSKGel guard column SuperMP (PW)-H (4.6 × 35 mm, Tosoh Bioscience, Griesheim, Germany). The operation conditions were: Milli-Q water at 0.6 mL/min as the mobile phase, and the column worked at 40 °C. Poly(ethylene oxide) from 2.36·10^4^ to 7.86·10^5^ g/mol (Tosoh, Tokyo, Japan) was used as standard.

#### 2.5.2. Fourier-Transform Infrared Spectroscopy

The lyophilized liquid extracts, alginates and microparticles of the brown seaweed *U. pinnatifida* were blended with potassium bromide (KBr) and dried using an infrared lamp (30 min). Several spectra were recorded at 600–1800 nm at 25 scans/min (Bruker IFS 28 Equinox equipment, OPUS-2.52), and the data acquisition was recorded using System 450-MT2. 

### 2.6. Production of Polymeric Microparticles

The microparticle production was performed using a mini spray-dryer B-290 (BÜCHI, Flawil, Switzerland) equipped with a standard cyclone and 1.5 mm nozzle. The test parameter was an inlet temperature of 103 °C, and the flow rate was always a fixed parameter. The operation conditions were 0.7 mL/min, where the pump worked at 20% (feed solution flow rate).

To produce the microparticles, a polymer with suitable properties (mannitol) was used as the carrier. Two extracts, obtained at two different temperatures, were selected according to the highest oligosaccharide (160 °C) and antioxidant fractions (220 °C). The microparticles were produced using a percentage of mannitol (5%) for the above-selected fractions, and after, a combination of them with mannitol was also tested according to Table 1.

### 2.7. Characterization of Microparticles 

#### 2.7.1. Yield of Production

The following Equation (2) was used to determine gravimetrically, the production yield (%):

(2) $$Production\;yield\left(\%\right)=\frac{mg\;microparticles\;recovered}{mg\;extract\;+\;mg\;mannitol}\cdot 100$$

#### 2.7.2. Scanning Electron Microscope

The shape of the microparticles was also studied using scanning electron microscopy (SEM, JEOL JSM6010LA, Tokyo, Japan). Before analysis, the polymeric microparticles were covered by a gold layer (15 nm), and then images at different scales (×500 and ×2000) were obtained. 

#### 2.7.3. Size of Microparticles

The assessment of the size of microparticles was studied using the SEM images, applying a special software, ImageJ. This parameter was read manually, up to 100 measures per formulation, to evaluate the size distribution of the samples to study.

### 2.8. Rheological Measurements

Steady-state shear viscosities of the extracted alginates and formulated particulate systems were measured on an MCR 302 controlled-stress rheometer (Paar Physica, Graz, Austria) at 25 °C. The selected geometry was a sand-blasted parallel plate of 25 mm diameter, operating at a 0.5 mm gap. The apparent viscosity of the prepared aqueous dispersions (2%, *w*/*w*) was followed by 0.1 to 100 1/s shear rates. The picked biopolymer amount is within the range of those commonly employed for natural sources in the food field [31]. The thixotropy phenomenon was evaluated by monitoring the forward and backward steady-state shear flows. It should be noted that light silicon oil was employed to cover the edges of the tested dispersions that were resting in the measuring geometry for 15 min before the rheological measurements to allow the thermal and structural balance of the dispersions. 

### 2.9. Statistical Analysis

All measures were statistically analyzed using one-factor analysis of variance, ANOVA. A post hoc test (Scheffé) was conducted to distinguish average data with different means, employing PASW Statistics software (v.22, IBM SPSS Statistics, New York, NY, USA). A confidence degree of 95% (*p* < 0.05) was employed.

## 3. Results and Discussion

### 3.1. Extraction Process

**Figure 1:**
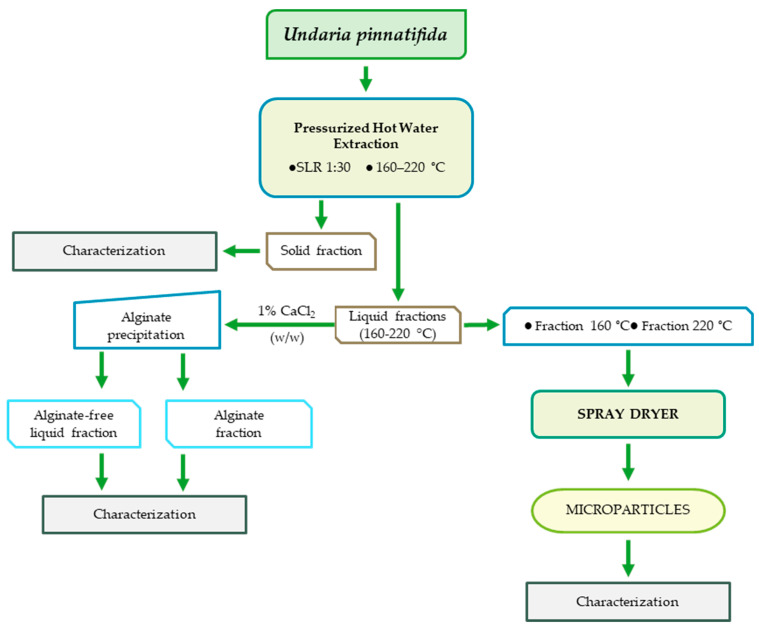
General scheme of the extraction using *U. pinnatifida* as raw material and the production of microparticles by spray–drier using mannitol as a carrier.

Based on previous works, the extraction technology proposed in this work was pressurized hot water extraction (Figure 1) [19,20,32]. The extraction temperatures, from 160 °C to 220 °C, were selected because other works found the maximum recovery of fucoidan at 170 °C, and for the antioxidant fraction, it was at 220 °C. In this context, the recovery of the bioactive compounds from edible brown seaweed *U. pinnatifida* to formulate polymeric microparticles with potential application in the food field is presented in the scheme below. This design focused on the eco-friendly process and zero waste, analyzing every fraction recovered and maintaining the circular bioeconomy concept.

**Figure 2:**
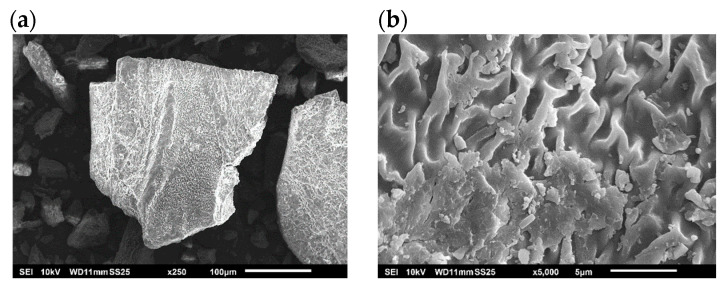

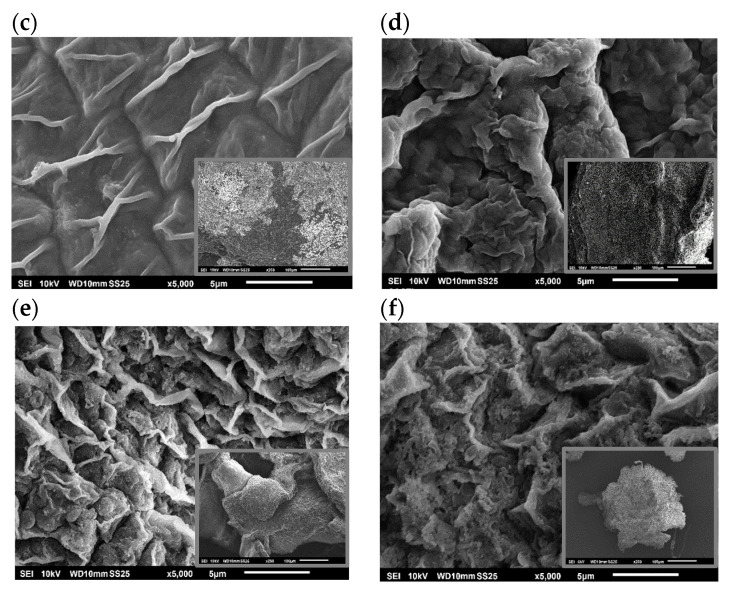
Scanning electron microscope images: (**a**,**b**) *U. pinnatifida* at different magnitude, solid residues after pressurized hot water extraction at (**c**) 160 °C, (**d**) 180 °C, (**e**) 200 °C and (**f**) 220 °C. All samples were presented at × 5000 and × 250.

The effect of the extraction process on the morphology of the raw material is presented in Figure 2. The surface of the seaweed was observed using scanning electron microscopy. The action of the treatment at temperatures from 160 to 220 °C in the cell wall of the alga was visible. The images show notable changes in the structure of the cell wall using different magnitudes (×500 and ×2000). Pressurized hot water extraction was also tested for the edible brown seaweed, *Himanthalia elongata*, observing similar changes in the surface of the alga [32]. Different eco-friendly extraction processes, such as microwave or ultrasound and their combination, were applied to the brown seaweed, *Ascophyllum nodosum*. The surface of this seaweed was also affected by extraction protocols, using water as a solvent [33]. This behavior, using several brown seaweeds, reveals the effect of the extraction technologies and their action of them in the cell wall to achieve bioactive compounds.

#### 3.1.1. Solid Residues Fractions Characterization

After the pressurized hot water extraction, the solid residues obtained at 160, 180, 200 and 220 °C (Figure 2) were analyzed and characterized; the results are shown in Table 2 [19]. The results exhibited similar behavior for the temperatures ranging from 160 °C to 220 °C, indicating an analogous effect in the extraction using pressurized hot water and microwave-assisted technologies. Other work, where the extracts from marine biomass were obtained by hot water extraction, has reported the composition of the solid residue. In this case, the main minerals were Ca, Mg and Na, finding lower values than those presented here [34]. This could be due to the severity of the extraction process; hot water extraction at atmospheric conditions (100 °C) is smooth in comparison with pressurized hot water extraction (160–220 °C). The cell wall will be affected with more impact, helping to release high-value compounds from this raw material. Additionally, the value of the HHV was evaluated, and we found a range of values from 16,665 to 22,803 kJ/kg. In the present work, the values obtained for the residue of the brown seaweed were higher in comparison to the values found for brown and red seaweeds, around 15,000–16,000 kJ/Kg [23,35]. Similar values were observed for other wastes, such as oil, brewing industries or compost, being in the range of around 16,000–20,500 kJ/kg [36]. 

#### 3.1.2. Liquid Fractions Properties

**Figure 3:**
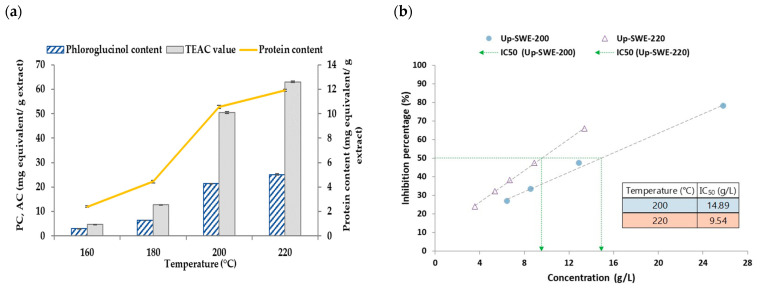

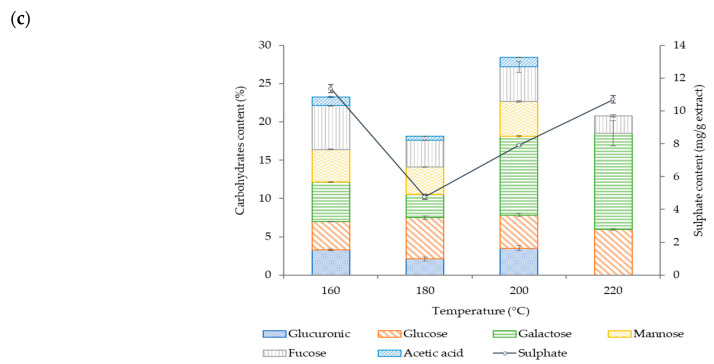
(**a**) TEAC value, phloroglucinol and protein content, (**b**) DPPH, (**c**) carbohydrate and sulphate content results of the liquid fractions obtained at 160–220 °C after pressurized hot water extraction of *Undaria pinnatifida* (Up).

Figure 3 represents the results obtained for the extracts recovered after pressurized hot water extraction at 160–220 °C from *Undaria pinnatifida*. The results for the phloroglucinol content, TEAC value and protein content exhibited a similar behavior where the maximum, in all cases, was obtained at the maximum temperature tested (220 °C). Other work, where the extraction was performed using different percentages of ethanol up to 200 °C, reported that the antioxidant activity and the phenolic content decreased, with the maximum recovery at 150 °C [11]. This tendency could be due to the solvent agent used for extraction and the employed technology. Other authors, Gan et al. (2022), found comparable results using *U. pinnatifida* as a raw material; in this case, values of around 1 mg/g extract for the extraction at 210 °C were obtained [37]. The protein content was evaluated in another work by Neri et al. (2019) using different samples from South Korea. The water-soluble protein values were consistent with those from this work [38]. The content of phloroglucinol was higher in this study compared with others. They found the maximum at 210 °C (0.99 mg/g dry weight), while the value obtained in the current work, at 160 °C, was close to 2.99 mg/g dry weight [37]. The DPPH inhibition percentages of the samples produced at 200 and 220 °C were studied, achieving IC_50_ of 9.5 g/L; however, such values are low compared to standard antioxidants. The carbohydrate and sulphate contents of the samples were assessed (Figure 3c). Different eco-friendly treatments have been reported to recover the fucoidan fraction, searching for a commitment between the fucose and sulphate contents [5]. The fucose value was maximum for the fraction obtained at 160 °C, with a value close to 6%, and also, in the case of the sulphate, it was 11%. According to these results, this is the fraction where the highest fucoidan content was achieved. Other works found the maximum fucose at 180 °C using other brown seaweed as the raw material while using the same extraction technology, where the value obtained was 9.43. However, the relation between the content of fucose and sulphate was maximum at 160 °C [32].

### 3.2. Structural Profiles

**Figure 4:**
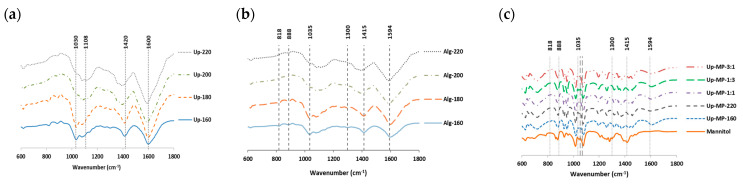
FT−IR spectra of the extracts obtained by pressurized hot water extraction: (**a**) the alginates precipitated, (**b**) the microparticles (MP), (**c**) formulated with extracts of 160 and 220 °C and its mixtures (3:1; 1:3; 1:1, 160 °C and 220 °C, respectively).

The FT-IR spectra of the extracts (Figure 4a) and alginates obtained by pressurized hot water extraction at different temperatures from *U. pinnatifida* are presented in Figure 4. The bands were associated with different functional groups; the band observed at 1030 cm^−1^ was ascribed to C-O stretching, whereas the peak at 1420 cm^−1^ was attributed to O-H bending. Furthermore, other authors consider these bands to indicate the presence of the saccharide fraction (C-H). Another band, found at 1600 cm^−1^, suggested the presence of C=C stretching in α, β-unsaturated ketone [39,40]. Similar bands were also reported in other works, where extracts from brown algae were characterized [41]. In general, the bands associated with the extracts obtained after pressurized hot water extraction were stronger at temperatures above 160 °C. The effect of the operational conditions of the eco-friendly process could be responsible for this appearance in the spectra. 

The alginates spectra were also studied (Figure 4b). The peaks found at 1035 cm^−1^ have been attributed to the C-O group, while the range of bands from 1400 to 1428 cm^−1^ was associated with the C-OH deformation vibration with the involvement of the symmetric stretching vibration of O-C-O. The peaks close to 1600 cm^−1^ have been suggested for O-C-O carboxylate asymmetric stretching [42]. Other works where the alginate bands from brown algae were characterized were consistent with these results [43]. To sum up, these bands demonstrated the precipitation of the alginate fraction at the different tested temperatures. Furthermore, at 200 and 220 °C, a peak at around 1100 cm^−1^ was identified. This band could be due to the severity of the extraction process at the highest tested temperatures. 

Additionally, the molar mass distribution profile of the extracts is represented in Figure S1. The molecular weight of the samples obtained at 160 and 200 °C showed a profile higher than the standards, whereas the extract recovery at 220 °C presented a profile near 786,000 Da. On the other hand, the sample obtained at 180 °C exhibited a maximum peak between 580,000 and 786,000 Da. In the present work, the profiles showed a large molecular weight of the extracts, being a key factor in terms of activity and applications. Whenever smaller molar mass distributions are required, an option could be applying other extraction technology to create a sequential treatment to promote the breakage of the molecules. Other authors, using the same raw material but different extraction technology (microwave heating), found differences in the profiles at the different temperatures (120–220 °C), where two fractions (small and large molecular weights) were observed [19]. Neupane et al. (2020), working with different brown algae, found differences in the profiles depending on the order (fucales, laminarales and ectocarpales) [44].

### 3.3. Production of Polymeric Microparticles

Based on the results obtained in the characterization of the fractions recovered by pressurized hot water extraction, two liquid phases were selected: 160 and 220 °C. These fractions have an important role because the maximum of the fucoidan was found at 160 °C, and the maximum of antioxidant activity and the phloroglucinol content was achieved at 220 °C. The microparticles were produced using mannitol as a carrier, and different parameters were evaluated.

The microparticles’ yield production for the extracts obtained at 160 and 220 °C was studied, achieving a value close to 75% (*w*/*w*) for the particles formulated with the extract recovered at 220 °C (Table 3). Focused on the application of these polymeric microparticles, searching for the optimal fucoidan and antioxidant fractions ratio (*w*/*w*) was addressed. In this case, the 1:1 ratio led to optimal performance (75%). This magnitude was similar to that obtained when the microparticles were exclusively formulated with extracts produced at 220 °C. These results allow for the enhancement of the bioactive composition without jeopardizing yield production. This action could improve the biological activity of the components of this edible seaweed, presenting a potential for food applications. Other authors formulated microparticles for food applications using Arabic gum as a carrier, achieving a value of 67% of yield production [45]. 

**Figure 5:**
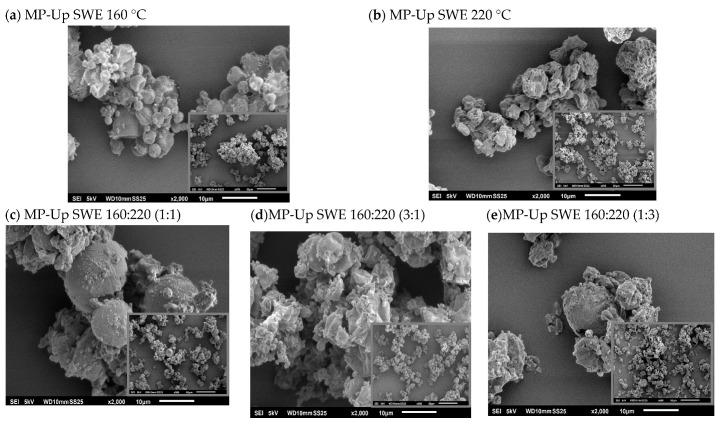
SEM images of the polymeric microparticles produced using mannitol and the extracts from *U. pinnatifida*: (**a**) 160 °C and (**b**) 220 °C. The ratios tested of both extract fractions (220:160) were (**c**) 1:1 and (**d**) 3:1, and (**e**) 1:3.

The morphological and size profile analyses were also performed to evaluate the shape and distribution of the microparticles and their behavior. Figure 5 represents the microparticles produced with the liquid extract at 160 and 220 °C, and also the different ratios of both fractions at different magnitudes (left: ×500, right: ×2000). In general, the images showed a heterogeneous shape, wide size distribution and aggregation. In other work, where microparticles for food application were developed, the spheric microparticles showed low heterogeneity, and this behavior could be due to the higher percentage of mannitol used [46]. The FT-IR spectra of the microparticles showed the bands associated with mannitol, demonstrating the blending of the extract and the mannitol polymer (Figure 4c).

**Figure 6:**
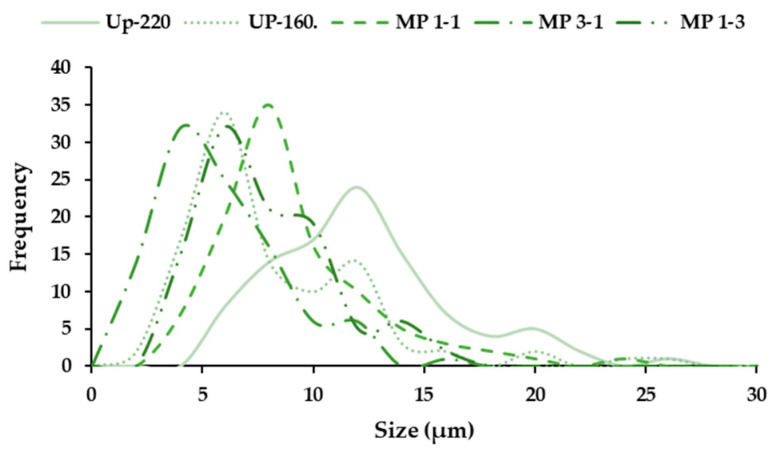
Size distribution of the polymeric microparticles formulated. Note Up: microparticles formulated with mannitol and the extracts obtained at 160 and 220 °C; the ratio 220:160 was evaluated at 1:1, 3:1 and 1:3.

The distribution of the particle size exhibited certain polydispersity, manifested in the images in Figure 6. The highest size and polydispersity were observed for the microparticles formulated with the extract produced at 220 °C, whereas the extract obtained at 160 °C showed a lower size. The mass ratios were also evaluated; the lowest polydispersity was observed for the 1:1 ratio, obtaining an intermediate size between 160 and 220 °C, being the lowest size for the ratio of 3:1. This behavior could be expected, observing the previous results for both extracts. Other authors found different size particle distributions using different biopolymers [47]. In future work, a further evaluation of the behavior of other polymers could be suitable.

### 3.4. Rheological Measurements

**Figure 7:**
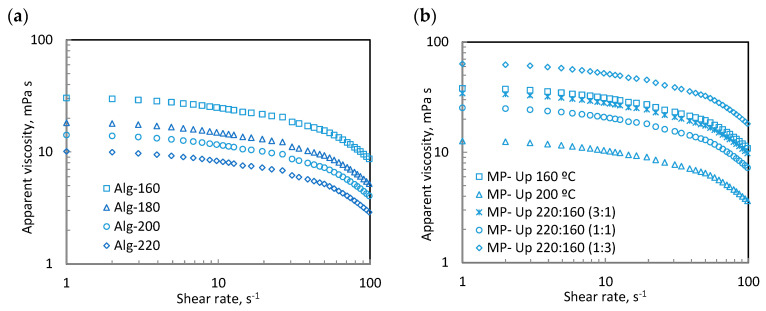
Flow curves for selected aqueous dispersions formulated with alginate (2%, *w*/*w*) in the (**a**) absence and (**b**) presence of microparticles (10%, *w*/*w*).

Figure 7a shows the apparent viscosity of the recovered alginates after hydrothermal treatment at different temperatures, which is critically relevant from the food processing and manufacturing point of view. In all cases, the apparent viscosity of the tested alginate dispersions (2%, *w*/*w*) measured at 25 °C exhibited non-Newtonian behavior. The hydrothermal treatment had a relevant impact on the apparent viscosity values, decreasing with increasing processing temperature. The highest apparent viscosities were identified for the alginates isolated after the mildest hydrothermal conditions (160 °C), which agrees with the highest molecular weight profiles found in the high-performance size-exclusion chromatography measurements since higher molecular weights can involve higher flow resistance, as previously reported for other biopolymers [48]. These tendencies and magnitudes are consistent with the flow curves reported in the literature for the alginates extracted from different brown seaweeds using selective solvents [49,50].

Figure 7b presents the apparent viscosity of the corresponding formulated microparticles. The presence of microparticles in the above aqueous solutions did not modify the non-Newtonian behavior, although it enhanced the apparent viscosity values over the tested shear rate range. The incorporation of microparticles involved competing with biopolymers for water and high flow resistance, as reported for other similar systems [50]. The highest viscous values were identified for MP-Up 220:160 (1:3), followed by MP-Up 160 °C, whereas the lowest values were observed for MP-Up 200 °C. 

It should be highlighted that thixotropic behavior was identified neither in the dispersions in the presence nor absence of microparticles at selected experimental conditions, with the subsequent profit for its final application.

## 4. Conclusions

The bioactive compounds from *U. pinnatifida* edible seaweed were obtained by subcritical water extraction and two fractions with potential interest were recovered: one at 160 °C, the maximum in fucose and sulphate content, and the other at 220 °C, the maximum in phloroglucinol content and antioxidant features. Polymeric microparticles were formulated using the extracts obtained (160 and 220 °C) and mannitol as the carrier. The results of the formulations exhibited suitable viscous and bioactive properties to be applied in the food industry, providing particular features, such as antioxidant power. Further analyses of the bioavailability of the bioactive compounds embedded in the microparticles could be interesting for future works in order to enhance potential food applications.

## Figures and Tables

**Table 1:** Liquid–liquid relation (*w*/*w*) of the mixture of selected fractions with mannitol as the carrier.

Relation (*w*/*w*)	Extract 160 °C	Extract 220 °C
3:1	3	1
1:1	1	1
1:3	1	3

**Table 2:** Proximal composition of the solid residues of *Undaria pinnatifida* brown seaweed after pressurized hot water extraction at 160 °C, 180 °C, 200 °C and 220 °C.

	**160 °C**	**180 °C**	**200 °C**	**220 °C**
Ash *	18.50	13.43	8.05	6.48
Sulphate (%)	0.78 ± 0.01	0.65 ± 0.01	0.37 ± 0.01	0.25 ± 0.01
C (%)	40.45 ± 0.24	43.50 ± 0.19	50.64 ± 1.49	54.20 ± 0.15
Protein (%)	24.83 ± 0.34	27.49 ± 0.84	31.88 ± 0.95	29.86 ± 1.14
H (%)	6.06 ± 0.09	5.95 ± 0.01	6.66 ± 0.01	6.65 ± 0.06
N (%)	4.62 ± 0.06	5.11 ± 0.16	5.93 ± 0.18	5.55 ± 0.21
HHV (kJ/kg)	16,665.72 ± 110.75	17,876.38 ± 52.42	21,146.91 ± 719.21	22,803.15 ± 70.22
Mineral **	(mg/kg)			
Na	101,812	98,203	104,423	102,408
K	99,979	97,246	102,996	99,294
Mg	13,103	13,376	15,094	15,032
Ca	5365	6111	8822	10,014
P	3331	3393	3933	4016
I	383	369	396	418
Fe	124.7	17.00	31.14	17.52
Zn	7.01	3.23	7.77	5.21
Cu	2.94	1.54	3.75	2.27
Cd	0	0	0.16	0.16

* Standard deviation lower than 5%. ** Standard deviation lower than 8%.

**Table 3:** Microparticles yield production using the extracts obtained by pressurized hot water extraction at 160 °C and 220 °C (160 and 220, respectively), and proportions tested (*w*/*w*).

	Production Yield (%)
MP-160	57.88 ± 1.57
MP-220	74.20 ± 3.55
MP-1:3 (220:160)	67.37 ± 0.57
MP-3:1 (220:160)	71.05 ± 0.45
MP-1:1 (220:160)	74.77 ± 0.79

## Data Availability

Data are contained within the article or supplementary material.

## Supplementary Materials

The following supporting information can be downloaded at: https://www.mdpi.com/article/10.3390/foods12091859/s1, Figure S1: Molar mass distribution of the extracts obtained from *U. pinnatifida* at 160, 180, 200 and 220 °C by pressurized hot water extraction.
