# Supplementary material for: Formulation of Polymeric Microparticles Using Eco-Friendly Extracted Crude Fucoidans from Edible Brown Seaweed Undaria pinnatifida
Source: Foods. 2023 Apr 29;12(9):1859. doi: 10.3390/foods12091859 (PMC10178044; doi:10.3390/foods12091859)
Supplement: Supplementary file 1 [file foods-12-01859-s001.zip › foods-2348615-supplementary.pdf]

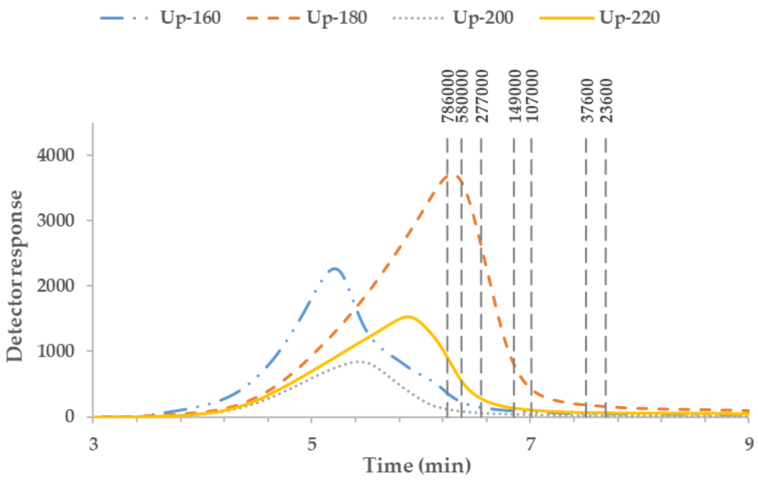

Figure S1. Supplementary. Molar mass distribution of the extracts obtained from *U. pinnatifida* at 160, 180, 200 and 220 °C by pressurized hot water extraction.
